# The Influence of Geographical Origin on Poplar Propolis Composition and the Impact of Human Microbiota

**DOI:** 10.3390/ph17060768

**Published:** 2024-06-11

**Authors:** Michał Miłek, Gabriela Franke, Monika Tomczyk, Miłosz Górecki, Olga Cwiková, Alžbeta Jarošová, Małgorzata Dżugan

**Affiliations:** 1 Department of Chemistry and Food Toxicology, Institute of Food Technology and Nutrition, University of Rzeszow, Ćwiklińskiej 1a St., 35-601 Rzeszow, Poland; mwesolowska@ur.edu.pl (M.T.); mdzugan@ur.edu.pl (M.D.); 2 Department of Food Technology, Faculty of AgriSciences, Mendel University in Brno, Zemědělská 1 St., 613 00 Brno, Czech Republic; gabriela.franke@mendelu.cz (G.F.); olga.cwikova@mendelu.cz (O.C.); alzbeta.jarosova@mendelu.cz (A.J.); 3 PROKIT—Miłosz Górecki, Świętokrzyska 25 St., Kazimierów, 05-074 Halinow, Poland; milosz.gorecki@prokit.pl

**Keywords:** propolis, chemical composition, polyphenols, antibacterial activity, gut microbiota

## Abstract

Ethanol extracts obtained from 13 poplar propolis samples originating from various European countries by traditional maceration were tested for total polyphenols, flavonoid content, and antioxidant activity. Moreover, the content of 18 polyphenolic compounds (from the group of phenolic acids and flavonoids) was determined using the HPLC method. The inhibitory effect of six selected extracts with the highest activity was assessed by well-diffusion method against five strains (*Bifidobacterium* spp., *L. rhamnosus*, *L. acidophilus*, *E. coli*, and *Bacteroides* spp.) of intestinal bacteria self-isolated from the faeces of obese probands with the use of selective media. It was found that the antioxidant activity of propolis varied depending on geographical origin and even among samples from the same region, which indicates that some other factors also influence propolis quality. The samples of different geographical origin varied mainly in the share of individual phenolic compounds, and it was not possible to find a characteristic marker of origin, excluding the galangin present in the Polish samples only. Assessing the inhibitory activity of propolis (in the range of 70 mg to 10 µg per mL) indicated that the concentration of 100 µg/mL was found as being safe for tested fecal bacteria (*Bifidobacterium* spp., *L. rhamnosus*, *L. acidophilus*, *E. coli*, and *Bacteroides* spp.). As no negative effect of low doses of propolis on the intestinal microflora was found, it can be suggested that its use in recommended doses brings only beneficial effects to the body.

## 1. Introduction

Propolis (or bee glue) is a resinous substance collected by honeybees (*Apis mellifera* L.) from plant buds, secretions, or resins found in the stems, branches, and leaves of various plants [1]. Considering that the main function of propolis is to support the sterility and health of the hive, the protective properties of the bioactive substances found in propolis may provide significant benefits to human health as well [2]. The mechanism by which propolis supports health seems to be related to its broad spectrum of action but is particularly based on its antioxidant, antimicrobial, anti-inflammatory, hepatoprotective, and immunomodulatory properties [3,4,5,6]. Therefore, propolis is widely used both in food and beverages, but also in cosmetics and pharmacology to improve human health and in the prevention of many diseases, among its other uses, such as inflammation, diabetes, heart disease, and even cancer [2,7,8,9]. Additionally, the literature describes the effect of propolis on various strains of viruses, such as the herpes simplex virus (HSV-1), the influenza virus (HIV-1), and even the SARS-CoV-2 virus [10,11,12]. Propolis also has the ability to inhibit the activity of various enzymes (acetylcholinesterase, lipoxygenase, α-glucosidase, xanthine oxidase, and hyaluronidase), which makes it a promising source of new active compounds used in the treatment of skin diseases, lifestyle diseases, and neurodegenerative diseases [13]. The beneficial properties of propolis are closely related to its rich chemical composition and the interaction of the individual components. According to actual literature, over 300 compounds have been described in propolis, including polyphenols, terpenoids, amino acids, sugars, steroids, minerals, and vitamins [1,4,14,15].

The composition of propolis is very variable and depends on various factors, such as the botanical and geographical origin (landforms), harvest time (month), season and climatic characteristics (weather), beekeeping practices (scraping from frames or propolis collector), and, finally, extraction method [8,16]. The basic composition of propolis is stable regardless of its origin and consists of about 50% resin, 30% wax, 5% pollen, 10% aromatic oils, and 5% other organic residues; however, the greatest variation is related to the presence or absence of selected polyphenolic compounds [6,17]. Flavonoids, such as galangin, apigenin, pinocembrin, pinostrobin, kaempferol, chrysin, and quercetin, as well as phenolic acids, including caffeic, ferulic, cinnamic, coumaric, and hydroxybenzoic, were determined in propolis samples from various geographic origins [10]. The most frequently identified compound in propolis from many countries, including Italy, Spain, Poland, and even India, is caffeic acid phenethyl ester (CAPE), characterized by its wide pharmacological effects, including anti-cancer activity [9,10]. Morphologically, there are three main types of propolis: temperate propolis, propolis from tropical regions, and propolis from the Pacific region [8]. In a temperate climate zone, bees collect propolis resin, mainly from the vegetative buds of poplars, primarily *Populus nigra* but also other *Populus* species including *P. tremula* (aspen), as well as from the leaf buds of *Betula* spp. and *Alnus* sp. (especially in Poland), which are also used by bees to produce propolis [8]. Poplar type propolis contains phenols typical of propolis from European poplar buds, i.e., flavonoids, phenolic acids, and their esters [18,19]. However, propolis is so botanically and geographically diversified and has such an extensive matrix that examining all the compounds is still a challenge for scientists.

The antibacterial effect of propolis is provided by polyphenolic substances, mainly flavonoids, which cause the inhibition of nucleic acid synthesis and DNA, cell wall, and membrane damage, as well as affecting the adhesion of bacteria to intestinal cells [20]. The bacteriostatic or bactericidal effect depends on the polyphenol structure and the particular strain of bacteria tested. It is estimated that only 5–10% of the total phenolic compounds are absorbed in the small intestine, while the remaining 90–95% are transported to the large intestine where they are biotransformed by the intestinal microflora. Studies by Ahn et al. [7] and Ozdal et al. [17] have demonstrated the degradation of propolis polyphenols at up to 80% and 87%, respectively. This claim is supported by many studies confirming the use of polyphenols as a substrate for colonic bacterial metabolism using enzymatic processes (deglycosylation, dehydroxylation, and demethylation). The resulting lower molecular weight metabolites (e.g., benzoic, phenylacetic, and phenylpropionic acids) have a bioactive effect on the organism and may exhibit anti-inflammatory activity [21,22,23]. Many studies confirm the metabolization of catechin and epicatechin by colonic bacteria; these are further absorbed by intestinal mucosal cells into the host bloodstream and subsequent metabolites are identified in blood plasma and urine [24,25,26].

While the antibacterial activity of propolis against pathogenic bacteria has been widely studied, the available literature completely lacks data on the impact of propolis on the microflora of the human gastrointestinal tract. Meanwhile, propolis is commonly used to treat infections and strengthen immunity by oral administration, which raises the question of whether such use does not strain the internal microflora. Thus, the main idea of this study was to evaluate the effect of poplar propolis extract on isolated human faecal bacteria during in vitro conditions. Considering the variability of the chemical composition of propolis, samples with extremely different levels of antioxidant activity and polyphenol profiles were selected at the preliminary stage from 13 propolis samples of distant geographical origin; thus, the comparative characterization of the chemical compositions of propolis from different parts of Europe was an additional result of the conducted research.

## 2. Results and Discussion

### 2.1. Variation in the Chemical Composition of Propolis Extract Resulted from Different Geographical Origin

Thirteen propolis extracts of different geographical origin were characterized in terms of the total content of polyphenols, flavonoids, and antioxidant activity, using three methods based on different mechanisms (Table 1). The study showed that the biological activity of propolis varied depending on its geographical origin. Moreover, the differences observed among samples from the same region indicate that the method of obtaining propolis influences its quality to a greater extent than its geographical origin does. The observed variability did not exceed 20%, except for the flavonoid content (39%), which seems to be unrelated to geographical origin. The samples with the highest antioxidant activity and polyphenol content included one from Ukraine (895), three from Poland (886, 887, 888), and one from Turkey (885). Among the tested samples of Polish propolis, three (75%) showed favorable values of the tested parameters, above the determined average. However, one sample (889) differed significantly from the others. Among the samples of different origins, those characterized by lower activity were the Bulgarian (890), Turkish (894, 897), and Ukrainian (896) samples.

The obtained results are in line with other studies where the high antioxidant activity of ethanolic extract of propolis was observed. For Polish propolis, in earlier studies, the polyphenol content ranged from 150.05 to 197.14 mg/g GAE, and total flavonoid content from 35.64 to 62.04 mg/g QE [19]. For Turkish propolis, the spread was even greater (TPC from 34.53 to 259.4 mg GAE/g and TFC from 21.28 to 152.56 mg CE/g) [1]. For propolis from various regions of Ukraine, the phenols content was 120.3 to 145.24 mg GAE/g, and the flavonoid fraction ranged from 53.9 to 82.71 mg QE/g [27]; similar values were reported for propolis from Romania [8]. Also, strong antioxidant activity, tested using various methods, is reflected in the available literature data for samples of similar geographical origin [8,27,28].

The results obtained using different methods were significantly correlated, as indicated by the values of Pearson correlation coefficients for DPPF-FRAP r = 0.89, DPPH-CUPRAC r = 0.88, and FRAP-CUPRAC r = 0.894. The total polyphenol content was significantly correlated with the flavonoid content TPC-TFC r = 0.832. A significant correlation was observed between antioxidant activity and polyphenol content, regardless of the research method used (r > 0.63), and between antioxidant activity and flavonoid content (r > 0.657), which indicates the pivotal role of these components in creating the antioxidant activity of the tested samples.

### 2.2. Variation in the Polyphenolic Profile of Propolis Extract According to Geographical Origin

The content of individual polyphenolic compounds in the extracts of the tested propolis was determined using the HPLC-DAD method (Table 2). Comparative analysis showed large differences in the content of individual polyphenolic ingredients depending on the origin of propolis. Based on the analysis of 13 samples, the average composition of the polyphenol fraction of propolis was established, and indicated that its active ingredients include phenolic acids (mainly caffeic, p-coumaric, and ferulic acid) and flavonoids (mainly chrysin, pinobanksin, and pinocembrin, as well as unidentified flavanones). This observation is in line with the literature data that indicate the presence of these compounds as typical for European poplar propolis [29,30,31].

The variability between samples of different geographical origin was mainly manifested in the different shares of individual compounds, and it was not possible to find characteristic marker compounds for samples from individual countries. For example, Polish propolis was characterized by the presence of an increased number of phenolic acids including, mainly, p-coumaric, caffeic, ferulic, and benzoic. However, the sample of Polish origin with the lowest activity contained mainly p-coumaric acid and a derivative of caffeic acid, while the content of other phenolic acids was lower than average. Among the flavonoids, Polish propolis was especially abundant in galangin, kaempferol, apigenin, and chrysin, while it contained less pinobanksin. Importantly, only in Polish propolis was galangin aglycone detected. This compound seems to be a promising origin marker. Two compounds from the flavanone group were also determined that could not be identified based on comparison with standards. These may include, for example, pinostrobin, the presence of which in European propolis is confirmed by the literature data [16,29].

**Figure 1:**
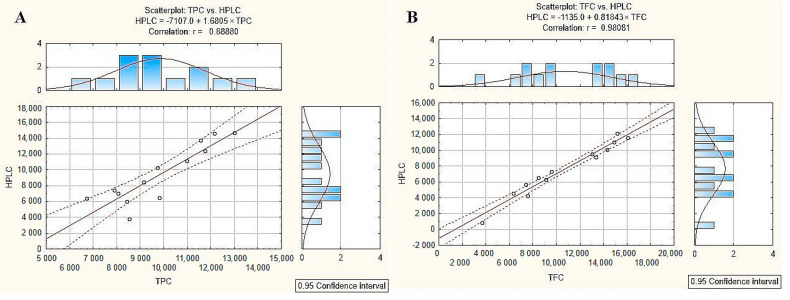
The correlation plots of total phenolic (**A**) and flavonoid (**B**) contents obtained by colorimetric and HPLC methods. Dashed lines indicate 0.95 confidence intervals.

The results of the total phenols and flavonoid content obtained using colorimetric and HPLC-DAD methods were compared. The distribution of the results is shown in Figure 1. A good correlation of the results was obtained, especially in the case of flavonoids (Pearson’s coefficient r = 0.981), whereas polyphenols were less correlated (r = 0.889); however, it should be remembered that the Folin—Ciocalteu method is less specific for determining polyphenol compounds.

Based on the analysis of 13 samples, the average composition of the polyphenol fraction of propolis was established, and indicated that its active ingredients include phenolic acids (mainly caffeic, p-coumaric, and ferulic acid) and flavonoids (mainly chrysin, pinobanksin, and pinocembrin, as well as unidentified flavanones). This observation is in line with the literature data that indicate the presence of these compounds as typical for European poplar propolis [29,30,31].

**Figure 2:**
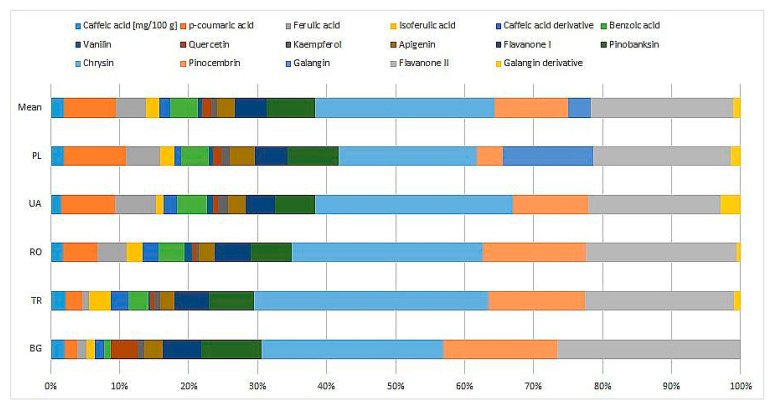
Average polyphenolic profiles for samples of different origins compared to the average profile of all tested samples. The shares of individual compounds are shown as percentages of the total sum of polyphenols determined by HPLC.

The compositions of the tested propolis samples were varied and the resulting re-sponse of the bacteria to the presence of these substances is difficult to interpret. Some in-teractions can be inferred from the available studies, for example, the inhibitory effect of some flavonoids (chrysin and pinobanksin) on the growth of beneficial bacteria [32]. In our study, positive correlations were observed between the detected concentrations of flavanone I and II, chrysin, and pinobanksin and the inhibition effect on *L. rhamnosus* (r = 0.93, r = 0.89, r = 0.87, resp. r = 0.85; *p* < 0.05). The averaged polyphenol profiles for the samples from the analyzed countries compared to the overall average are shown in Figure 2. Generally, the polyphenol composition was similar, with some differences that are most evident in the content of phenolic acids (Poland and Ukraine) or flavonoids (Bulgaria and Turkey). For Bulgarian propolis, a characteristic predominance of quercetin content over kaempferol was observed, in contrast to the other samples.

**Figure 3:**
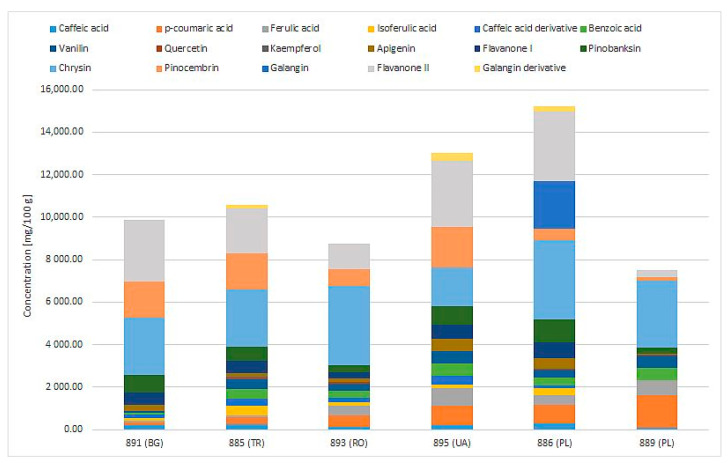
Polyphenolic profiles of propolis samples selected for in vitro microbiological study.

Based on differences in the obtained polyphenolic profiles, five samples were selected for the next step of analyzing the impact on the human microbiota (Figure 3). Samples with the highest content of polyphenols were selected from each country tested, and for comparison, the propolis sample with the lowest levels of these compounds (889, Poland) was applied. The specific feature of both Polish propolis samples was a lower pinocembrin content and the presence of galangin.

### 2.3. The Effect of Propolis on Fecal Bacteria In Vitro

Table 3 shows the antibacterial activity of the propolis extracts against bacteria obtained from the gut microbiome of probands. The concentration of the propolis samples tested at which they no longer showed an inhibitory effect against the micro-organisms tested was 100 µg/mL, except for sample 886, for which the inhibition of *L. rhamnosus* and *Bacteroides* spp. was observed at this concentration.

The compositions of the tested propolis samples varied and the resulting response of the bacteria to the presence of these substances is difficult to interpret. Some interactions can be inferred from the available studies, for example, the inhibitory effect of some flavonoids (chrysin and pinobanksin) on the growth of beneficial bacteria [32]. In our study, positive correlations were observed between the detected concentrations of flavanone I and II, chrysin, and pinobanksin and the inhibition effect on *L. rhamnosus* (r = 0.93, r = 0.89, r = 0.87, resp. r = 0.85; *p* < 0.05) only.

**Figure 4:**
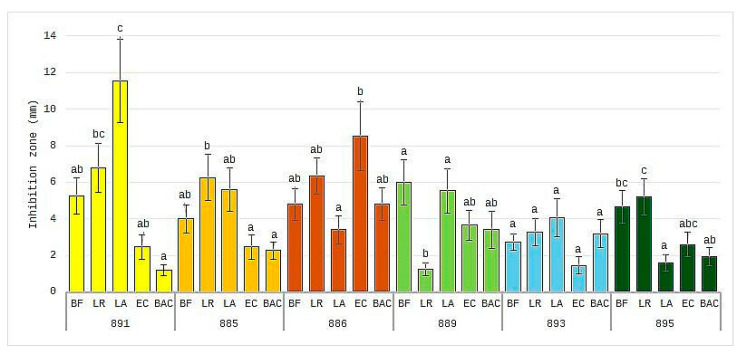
Diameter of inhibition zone (after subtraction diameter of well; in mm) of strains: *Bifidobacterium* spp. (BF), *Lactobacillus rhamnosus* (LR), *Lactobacillus acidophilus* (LA), *Escherichia coli* (EC), *Bacteroides* spp. (BAC) obtained after incubation with ethanolic extracts of propolis (EEP) regardless of the concentration. a–c letters mark significant differences (*p* < 0.05) between inhibition of strains by each sample of EEP.

The evaluation of the antibacterial effect of propolis regardless of its concentration (Figure 4) allowed for ranking the tested samples in terms of their impact on individual bacterial strains. The antioxidatively strongest propolis samples, 891 (BG) and 886 (PL), showed a tendency toward different effects: the first one strongly inhibited the growth of probiotic bacteria (*Bifidobacterium* spp., *L. rhamnosus*, and *L. acidophilus*), and the growth of *E. coli* and *Bacteroides* spp. less strongly, whereas the second one, on the contrary, tended to inhibit the growth of all bacteria except for *L. acidophilus*. This effect may be related to the different chemical profiles of the samples. Since a significant difference was found concerning the presence of galangin in Polish propolis as well as the high content of quercetin in Bulgarian propolis, it can be suspected that these flavonoids may play an important role in shaping the antibacterial properties of propolis. These results are confirmed by the study by Omidi et al. [33], who observed no antibacterial effect on *L. acidophilus* of the matrix with pure galangin.

In the case of *Bacteroides* spp., various inhibition effects among samples of propolis were detected. Recent genomic methods contributed to our understanding of the unique adaptive nature of the *Bacteroides* species. Bacteria of the *Bacteroides* genus are considered a reservoir of genes for antibiotic resistance [34].

However, taking into account the results from our previous work, where the MIC value determined for pathogenic bacteria (*E. coli* and *Staphylococcus* spp.) was 24–780 µg/mL [9], the applied concentrations of 100 µg/mL (0.01%) and 10 µg/mL (0.001%) seem to be safe for human microbiota. Assuming a daily intake of max. 30 drops of standard propolis extract, which corresponds to approximately 0.08 g of dry extract, and taking into account its dilution in the digestive content, the exposure of the microbiome in the intestine should not exceed the concentration of 0.01% tested in in vitro tests. It can be assumed that such therapy will be effective against pathogenic bacteria and, at the same time, safe against beneficial intestinal bacteria.

Many studies show an inhibitory effect of phenolics on pathogenic bacteria but a stimulatory effect on commensal bacteria of the digestive tract [35]. However, this area is still under-researched, and long-term in vivo research is needed to confirm the aforementioned hypothesis of a positive effect on beneficial intestinal bacteria. Some attempts were also made for single flavonoids, including quercetin and galangin, which were recognized as prebiotics [36,37].

In the study of Ferreira de Brito et al. [38], nine samples of Brazilian propolis extract were tested. An inhibition effect against *L. rhamnosus* was detected at concentrations of 500–1000 μg/mL and against *Staphylococcus aureus* and *Salmonella typhymurium* at 125–500 μg/mL and 250 μg/mL, respectively. Quercetin belongs to the important antibacterial substances of propolis and can be used for its effect in the prevention of bacterial diseases of the digestive tract of animals. The application of 0.4 g quercetin/kg to feed significantly increased (*p* < 0.01) the numbers of *Bifidobacterium* spp. and *Lactobacillus* spp. in the digestive tract of broilers, while the numbers of the pathogenic bacteria *P. aeruginosa, S. enterica,* and *S. aureus* decreased (*p* < 0.05, resp. *p* < 0.01) [39]. In the case of bifidobacteria, regression analysis confirmed the same effect, with a rising dose of quercetin increasing the number of bifidobacteria in the intestine (*p* < 0.05) [40]. The same effect was reported in a study of Kačániová et al. [41], who tested the addition of propolis extract to chicken feed. The numbers of lactobacilli in the digestive tract of chickens compared to the control group were increased in all experimental groups (100–800 mg of propolis/kg of feed).

A last study by Garzarella et al. [42] monitored the amount of polyphenols metabolized during a simulated digestive process. During the simulated digestion process and subsequent fermentation by the intestinal bacteria of healthy people, the polyphenol content was metabolized from the original 70.0 g of gallic acid equivalents/kg propolis to 2.5 ± 0.4 g of gallic acid equivalents/kg propolis after simulated digestion. However, the most significant decrease was observed in the case of the mixed bacterial microflora of obese people, where a value of up to 2.1 ± 0.2 g gallic acid equivalents/kg propolis was observed. In our study, bacterial strains from obese subjects were obtained (BMI > 25 kg/m^2^); therefore, based on these results, it can be expected that the metabolism of phenolic compounds was at a higher level and, thus, the inhibitory effect was at a lower level than it would be in healthy individuals.

## 3. Materials and Methods

### 3.1. Chemicals

Chemicals (2,2-diphenyl-1-picrylhydrazyl, 2,2-azino-bis(3-ethylbenzothiazoline-6-sulfonic acid; 2,4,6-Tris(2-pyridyl)-s-triazine, neocuproine, copper(II) chloride, ammonium acetate, sodium carbonate, ferric chloride), reagents (Folin–Ciocalteu reagent), HPLC standards (gallic acid, caffeic acid, ferulic acid, benzoic acid, p-coumaric acid, vanillin, apigenin, quercetin, kaempferol, galangin, chrysin, pinobanksin, pinocembrin) were obtained from Sigma Aldrich (Saint Louis, MO, USA), and solvents (ethanol, acetonitrile, formic acid) were purchased from Avantor Performance Materials Poland SA (APM, Gliwice, Poland).

### 3.2. Media for Fecal Bacteria Isolation and Antibacterial Activity Testing

These included Bifidus selective medium agar with BSM Supplement (Sigma Aldrich, Saint Louis, MO, USA); MRS agar, *E. coli*/coliforms agar, Plate count agar (PCA), and Ringers solution tablets (¼ strength) (Neogen, Lansing, MI, USA); clindamycin hydrochloride, vancomycin, Bacteroides Bile esculin agar with Bacteroides Selective Supplement, and Nutrient Broth Peptone (Himedia, Mumbai, India); Mueller Hinton II agar and Gentamicin disk (30 μg) (Liofilchem, Roseto degli Abruzzi, Italy); and 70% ethanol (Lach-Ner, Neratovice, Czech Republic).

### 3.3. Propolis Samples and Extract Preparation

Thirteen samples of poplar propolis with various geographical origins were provided by the company PROKIT—MIŁOSZ GÓRECKI (Kazimierów, Poland), which specializes in purchasing raw propolis directly from beekeepers. The set included: 4 Polish (sample codes: 886, 887, 888, and 889), 3 Turkish (879, 885, and 894), 2 Romanian (892 and 893), 2 Ukrainian (895 and 896), and 2 Bulgarian (890 and 891) propolis samples. All samples showed organoleptic features (color, smell, texture) typical for poplar propolis.

For each propolis sample, an extract was prepared strictly by the same maceration procedure in duplicate. Specifically, 10 g of each propolis sample was poured with 100 mL of 70% ethanol in a glass bottle with a screw cap and shaken in the dark for 30 min at 400 rpm. After this time, propolis was macerated in dark at room temperature (20 ± 2 °C) within 5 days. Every day samples were manually shaken. Then, extract was filtered through a filter paper and subjected to the two-step condensation process, including ethanol evaporation (RVC 2–18 CDPlus, Martin Christ, Osterode am Harz, Germany) and freeze-drying (Alpha 1–2 LD plus, Martin Christ, Osterode am Harz, Germany). The obtained dry extract was dissolved in 70% ethanol (100 mg/mL concentration) and used for the determination of antioxidant activity, total polyphenols and flavonoid content, chromatographic analyzes, and antimicrobial effect in vitro study.

### 3.4. Antioxidant Activity Assays

DPPH radical scavenging activity was measured based on the procedure described by Miłek et al. [9]. Briefly, 0.02 mL of appropriate propolis extract was added to 0.18 mL of 0.1 mM DPPH solution in methanol and left in dark for 30 min. Then, the absorbance was measured at 517 nm using a UV–VIS Spectrometer (EPOCH 2 microplate spectrophotometer, BioTek, Winooski, VT, USA). The results obtained were expressed as μmol of Trolox equivalents (TE) per 1 g of the dry weight of the extract based on the prepared standard curve (25–300 nmol/mL of Trolox solution in methanol).

FRAP (Ferric Reducing Antioxidant Power) assay was also provided according to Miłek et al. [9]. Briefly, 0.02 mL of sample was mixed with 0.18 mL FRAP reagent consisted of 2.5 mL of a 10 mM 2,4,6-tripyridyltriazine (TPTZ) solution in 40 mM HCl, 2.5 mL of 20 mM FeCl_3_ and 25 mL of 0.3 M acetate buffer (pH 3.6), and the absorbance of the mixture was measured spectrophotometrically (EPOCH 2 microplate spectrophotometer) at 593 nm after 10 min of incubation at 37 °C against blank. A calibration curve was prepared for Trolox ethanol solution in the range 25–300 nmol/mL, and the results were expressed as µmol of Trolox equivalents (TE) per 1 g of the dry extract.

CUPRAC (CUPric Reducing Antioxidant Capacity) assay was performed according to Dżugan et al. [43]. Briefly, 10 μL of diluted propolis dry extract was mixed with 40 μL of CuCl_2_ (10 mM), 50 μL of neocuproine (7.5 mM), and 50 μL of ammonium acetate (1 M). The reaction mixture was then incubated at room temperature for 30 min and the absorbance was measured with a microplate reader (EPOCH2, BioTek, Winooski, VT, USA) at 450 nm. The results were expressed as Trolox equivalents per 1 g of dry extract based on a calibration curve (125–2000 nmol/mL).

### 3.5. Total Phenolic and Flavonoid Content Analysis

The total phenolic content (TPC) was also measured using the procedure described by Miłek et al. [9]. In summary, 0.02 mL of plant extract was mixed with 0.1 mL Folin–Ciocalteu reagent (10-fold diluted) and, next, 0.08 mL of 7.5% (*w*/*v*) Na_2_CO_3_ solution was added. After incubation at room temperature for 60 min, the absorbance was measured spectrophotometrically (EPOCH 2 microplate spectrophotometer) at 760 nm against the blank. TPC was calculated based on a calibration curve at the range 25–150 µg/mL prepared with gallic acid (GA). Results were expressed as mg of gallic acid equivalents (GAE) per 1 g of the extract.

The total flavonoid content (TFC) was assessed according to by Miłek et al. [9]. Briefly, 0.1 mL of the propolis solution was mixed with 0.1 mL 2% AlCl_3_ (in methanol). The reaction mixture was incubated for 10 min at room temperature until the reaction was complete. The absorbance of the solution was then measured at 415 nm with a microplate reader EPOCH 2 against methanol blank. The total content of flavonoids in the extracts was expressed in mg of quercetin equivalent (QE) per g of dry extract (mg QE/g). The results were calculated based on a calibration curve prepared for quercetin in the range 0–125 µg/mL.

### 3.6. HPLC Analysis

The content of selected polyphenolic components of the propolis extracts have been quantified by HPLC-DAD method using Gilson HPLC System (Gilson Inc., Middleton, WI, USA). The analytical column (Poroshell 120, EC C-18, 4.6 × 150 mm, Agilent Technologies Inc., Santa Clara, CA, USA) has been applied. Before analysis, extracts were filtered through 0.22 μm syringe filters and diluted 5 times. A 10 µL injection was used, with a 1 mL/min flow gradient elution mode using 0.1% formic acid in distilled water (A) and acetonitrile (B). Gradient program with gradient: 0–1.5 min 10% B, 1.5–3 min 10–30% B, 3–9 min 30–50% B, 9–24 min 50% B, 24–29 min 50–100% B, 29–33 min 100% was applied and 10% B was used again to equilibrate the column. The components of the extracts were identified based on a comparison of UV-Vis spectra and retention times with standards. For quantitative analysis, the standard curve method was used for the following standards: caffeic acid, p-coumaric acid, ferulic acid, benzoic acid, vanillin, apigenine, quercetin, kaempferol, pinobanksin, pinocembrin, and galangin. For all compounds, calibration was linear, in the range of 12.5–250 μg/mL (R^2^ > 0.997). The results were expressed as mg per 100 g of propolis.

### 3.7. In Vitro Testing of the Propolis Effect on Fecal Bacteria

#### 3.7.1. Isolation of Intestinal Bacteria from Faeces

For isolation of five intestinal bacteria taxons: *Lactobacillus acidophilus*, *Lactobacillus rhamnosus*, *Bifidobacterium* spp., *Escherichia coli*, *Bacteroides* spp. the samples of human faeces from obese subjects were obtained (women, aged in the range of 18–52 years, BMI > 25 kg/m^2^, without pharmacological treatment) were used. The method for isolation according Komprda et al. [44] with a little modification was applied. 0.5 g of faecal sample was homogenized with 4.5 mL of Nutrient Broth Peptone and pre-cultivated 4 h at 37 °C. After that, decimal dillutions were performed and following bacteria were isolated: *Bifidobacterium* spp. on Bifidus selective medium (BSM) agar at 37 °C after 72 h of anaerobic cultivation; *Lactobacillus acidophilus* and *Lactobacillus rhamnosus* on MRS medium adjusted with clindamycin hydrochloride 0.5 mg/mL, resp. vancomycin 2 mg/mL after 72 h of anaerobic cultivation at 37 °C; *Escherichia coli* on *E. coli*/coliforms agar after 48 h of aerobic cultivation at 37 °C; *Bacteroides* spp. on Bacteroides Bile esculin agar base adjusted with supplement after 72 h of anaerobical cultivation at 37 °C (colonies were harvested regardless esculin hydrolysis reaction).

After isolation on the selective media bacteria were purified on non-selective agar PCA and cultivated at the same conditions. The fresh purified cultures were used for isolation of DNA by NucleoSpin Tissue (Machery Nagel, Dueren, Germany) according to the producer instructions. The samples of purified bacterial DNA were identified by polymerase chain reaction (PCR) using primers selective for the following species, targeting marker DNA sequences deposited in the GenBank (GenBank, Bethesda, MD, USA): for *Bifidobacterium* spp., accession number AF261684; for *Lb. acidophilus*, U32971; for *Lb. rhamnosus*, U32966; and for *E. coli*, J01636. *Bacteroides* spp. were identified as typical colonies according the manufacturer’s instructions for the selective medium.

#### 3.7.2. Well-Diffusion Method

Antimicrobial activity of propolis ethanolic extracts by well-diffusion method was performed according to protocol CLSI M7 [45] and M11 [46]. Bacterial cultures were grown on PCA agar for 18 h at 37 °C aerobically, resp. anaerobically. The cell suspensions were prepared with sterile Ringer’s solution to the 0.5 McFarland standard. Inoculation of Petri dishes with Mueller Hinton II agar were performed by sterile swabs immersion into cell suspensions. Using a cork borer (Fisher Scientific, Sunnyvale, CA, USA), the wells (in diameter 6 mm) were made and 30 µL of tested propolis solutions were pipetted into the wells. The antimicrobial effect of propolis solutions on bacterial cultures were evaluated after 24 h at 37 °C aerobically, resp. anaerobically. Gentamicin disk (30 μg) as a positive control and 70% ethanol (Lach-Ner, Czech Republic) as a negative control were used. The diameter of inhibition clear zones (in mm) was measured around the wells. All tests were carried out in triplicate.

### 3.8. Statistical Analysis

All analyses were performed in triplicates and the results are expressed as mean ± standard deviation. The results were subjected to one-way ANOVA and the significance of differences was determined based on Tukey’s test (*p* = 0.05). Correlations between obtained results were calculated using r Pearson’s coefficients. All calculations were made using the Statistica 13.3 software (StatSoft, Tulsa, OK, USA).

## 4. Conclusions

The study confirmed that the levels of antioxidant activity and the polyphenolic profiles of European poplar propolis show diversification depending on the geographical origin of the sample. The differences observed for samples from the same region suggest the great impact of other factors. Unfortunately, it was not possible to establish universal markers useful to determine the place of origin. The assessment of the impact of poplar propolis extracts (in the range of 70 mg to 10 µg per mL) on human microbiota indicates the concentration of 100 µg/mL as safe for all tested fecal bacteria. However, a significant correlation between phenolic compositions and an inhibitory effect was not found. It has been shown that appropriate dosing of propolis does not affect the intestinal microflora, which allows us to assume that there are only beneficial effects of the use of propolis extract in prevention and treatment. As such, research using propolis samples from various European countries was carried out for the first time, and, taking into account the complex and variable chemical compositions of propolis, further extensive research, including in vivo tests, is required.

## Figures and Tables

**Table 1:** Total phenolic and flavonoid contents as well as antioxidant activity evaluation using different analytical methods for 13 samples of propolis differing in geographical origin.

Sample	TPC [mg GAE/g]	TFC [mg QE/g]	DPPH [μmol TE/g]	FRAP [μmol TE/g]	CUPRAC [μmol TE/g]
890 (BG)	80.51 ± 4.44 ^a^	90.99 ± 4.78 ^c^	171.63 ± 7.41 ^ab^	284.49 ± 34.15 ^ab^	1412.82 ± 138.91 ^a^
891 (BG)	97.26 ± 5.65 ^bde^	130.48 ± 5.66 ^d^	207.48 ± 12.00 ^bdef^	354.36 ± 6.45 ^ce^	1874.36 ± 282.25 ^af^
894 (TR)	67.38 ± 4.72 ^a^	73.84 ± 2.88 ^bc^	183.95 ± 23.54 ^acdk^	330.16 ± 6.34 ^bcd^	1667.95 ± 109.62 ^acdi^
879 (TR)	78.97 ± 3.60 ^a^	84.48 ± 3.64 ^c^	201.38 ± 14.00 ^ae^	306.83 ± 30.30 ^ac^	1602.56 ± 193.59 ^ab^
885 (TR)	109.74 ± 8.33 ^b^	133.74 ± 5.03 ^df^	287.25 ± 7.69 ^l^	523.65 ± 26.99 ^g^	2685.90 ± 276.99 ^h^
892 (RO)	91.51 ± 7.75 ^aef^	95.83 ± 4.82 ^c^	236.47 ± 18.80 ^e^	328.73 ± 29.89 ^bcde^	1915.38 ± 362.21 ^ag^
893 (RO)	98.15 ± 5.22 ^bdf^	63.21 ± 2.54 ^b^	209.47 ± 5.29 ^bdeg^	364.21 ± 21.50 ^cf^	2132.05 ± 311.05 ^befgh^
895 (UA)	130.00 ± 5.45 ^c^	160.46 ± 9.34 ^e^	225.65 ± 11.79 ^ejk^	390.49 ± 25.75 ^ef^	2308.97 ± 99.88 ^defgh^
896 (UA)	84.42 ± 6.69 ^ad^	75.47 ± 2.95 ^bc^	167.27 ± 8.44 ^a^	278.03 ± 8.85 ^ab^	1671.79 ± 207.70 ^ae^
886 (PL)	121.48 ± 2.97 ^bc^	151.60 ± 7.81 ^e^	244.19 ± 12.96 ^fghij^	358.96 ± 33.66 ^ce^	2252.56 ± 180.99 ^bcefgh^
887 (PL)	115.78 ± 3.75 ^bc^	148.80 ± 10.69 ^ef^	222.41 ± 11.20 ^cei^	401.55 ± 24.78 ^ef^	2271.79 ± 235.66 ^efghi^
888 (PL)	117.52 ± 6.74 ^bc^	143.31 ± 7.10 ^de^	219.92 ± 11.79 ^ceh^	386.77 ± 13.80 ^def^	2021.79 ± 169.94 ^ah^
889 (PL)	85.41 ± 4.17 ^ad^	36.64 ± 1.59 ^a^	163.17 ± 14.01 ^a^	241.46 ± 0.57 ^a^	1676.92 ± 182.48 ^ae^
Min	61.98	34.81	148.98	241.13	1280.77
Max	136.30	167.02	293.10	545.34	2900.00
Mean	98.32	106.84	210.79	349.98	1961.14
SD	19.20	39.07	35.61	71.46	396.14
Variability [%]	19.53	36.57	16.89	20.42	20.20
F-value	35.42	135.74	21.26	28.17	7.67
*p*-value	0.000	0.000	0.000	0.000	0.000

BG—Bulgaria, TR—Turkey, RO—Romania, UA—Ukraine, PL—Poland; ^a,b,c,d,e,f,g,h,i,j,k,l^—means marked with different letters are significantly different at *p* < 0.05. Data are presented as mean ± SD (*n* = 3).

**Table 2:** The individual polyphenolic compound content in tested propolis samples (mg/100 mg).

		Polyphenol Content [mg/100 g]																	
		Caffeic Acid	p-Coumaric Acid	Ferulic Acid	Isoferulic Acid	Caffeic Acid Derivative	Benzoic Acid	Vanillin	Quercetin	Kaempferol	Apigenin	Flavanone I	Pinobanksin	Chrysin	Pinocembrin	Galangin	Flavanone II	Galangin Derivative	Sum of polyphenols
Propolis sample no. (country of origin)	890 (BG)	148.86 ± 11.22	181.47 ± 8.62	142.41 ± 18.53	82.13 ± 4.20	92.53 ± 7.08	72.80 ± 4.45	nd	240.80 ± 17.95	75.44 ± 22.95	188.87 ± 5.00	366.66 ± 31.11	688.68 ± 42.82	1873.22 ± 145.29	1155.22 ± 81.09	nd	1704.02 ± 136.13	nd	7013.11
	891 (BG)	210.87 ± 12.05	135.56 ± 14.14	82.39 ± 3.73	132.68 ± 13.60	140.46 ± 12.11	92.58 ± 13.72	nd	423.88 ± 32.97	99.86 ± 38.84	268.00 ± 5.88	599.16 ± 24.41	815.14 ± 29.98	2694.24 ± 100.37	1699.43 ± 17.82	nd	2893.38 ± 123.84	nd	10,287.63
	894 (TR)	127.81 ± 17.02	126.43 ± 34.37	58.71 ± 19.49	203.85 ± 27.46	127.35 ± 20.50	88.65 ± 28.87	12.45 ± 5.12	92.57 ± 7.59	64.44 ± 13.53	191.56 ± 11.05	298.86 ± 63.28	468.03 ± 42.33	2290.17 ± 208.16	739.58 ± 206.56	nd	1537.51 ± 352.02	nd	6427.97
	879 (TR)	153.43 ± 4.12	159.28 ± 10.23	75.61 ± 5.08	168.92 ± 5.27	143.99 ± 9.99	179.63 ± 2.11	13.81 ± 0.66	85.30 ± 3.27	62.41 ± 4.92	191.90 ± 2.32	364.34 ± 35.14	492.12 ± 27.07	2432.83 ± 142.29	1065.96 ± 41.07	nd	1715.16 ± 71.17	96.15 ± 12.81	7400.84
	885 (TR)	272.16 ± 6.54	302.83 ± 12.39	104.39 ± 10.69	433.07 ± 10.49	341.31 ± 9.76	458.91 ± 18.49	32.84 ± 1.95	nd	63.81 ± 4.09	144.26 ± 6.04	582.62 ± 25.43	675.30 ± 32.23	3728.38 ± 173.08	1697.07 ± 64.99	nd	2123.39 ± 20.86	141.93 ± 13.07	11,102.27
	892 (RO)	156.98 ± 9.17	206.40 ± 19.80	142.43 ± 16.47	173.07 ± 9.85	184.61 ± 8.85	227.68 ± 13.72	48.54 ± 6.86	95.69 ± 6.82	54.91 ± 22.21	167.20 ± 22.24	500.10 ± 20.00	563.59 ± 28.81	2310.67 ± 85.51	1457.63 ± 63.02	nd	2056.45 ± 90.63	94.55 ± 3.77	8440.43
	893 (RO)	112.47 ± 17.02	540.85 ± 51.52	482.88 ± 47.51	169.79 ± 28.36	175.53 ± 20.64	328.85 ± 33.85	113.71 ± 11.37	nd	nd	175.01 ± 11.37	270.26 ± 41.58	335.62 ± 51.79	1819.02 ± 561.25	781.53 ± 123.86	nd	1195.44 ± 173.84	nd	6500.96
	895 (UA)	191.50 ± 13.89	950.12 ± 50.39	810.50 ± 37.69	180.15 ± 14.75	400.27 ± 23.10	569.85 ± 24.30	40.66 ± 1.95	159.04 ± 10.14	179.10 ± 19.98	540.27 ± 11.80	683.40 ± 39.44	862.48 ± 50.05	3723.49 ± 117.67	1906.02 ± 86.07	nd	3115.29 ± 167.31	380.85 ± 11.04	14,692.99
	896 (UA)	122.90 ± 64.48	660.11 ± 273.44	429.33 ± 5.10	54.28 ± 7.02	nd	321.63 ± 6.40	139.57 ± 8.48	nd	108.51 ± 12.47	nd	190.22 ± 19.43	335.82 ± 38.95	2220.39 ± 348.32	350.84 ± 118.32	nd	837.42 ± 151.35	227.00 ± 21.54	5998.02
	886 (PL)	300.24 ± 5.54	847.40 ± 96.13	492.66 ± 55.14	299.82 ± 8.20	130.60 ± 9.90	379.81 ± 67.42	50.03 ± 6.47	202.44 ± 5.57	164.31 ± 22.85	470.48 ± 23.75	757.57 ± 7.56	1068.69 ± 25.30	3140.17 ± 166.06	574.12 ± 26.69	2201.42 ± 2.29	3301.33 ± 113.70	234.24 ± 6.30	14,615.33
	887 (PL)	251.81 ± 17.78	881.74 ± 21.56	515.37 ± 24.51	333.98 ± 27.94	148.32 ± 6.86	479.56 ± 21.30	77.81 ± 7.52	187.61 ± 16.85	163.82 ± 41.05	600.26 ± 31.72	674.49 ± 63.06	897.59 ± 100.91	3011.42 ± 213.99	497.22 ± 43.88	1933.68 ± 161.43	2883.25 ± 187.57	216.46 ± 15.16	13,754.39
	888 (PL)	254.47 ± 25.51	738.65 ± 38.42	454.18 ± 48.15	306.31 ± 31.70	139.60 ± 18.84	341.33 ± 29.98	76.12 ± 8.49	198.94 ± 19.80	171.21 ± 34.64	507.80 ± 23.33	687.67 ± 44.90	1048.45 ± 82.87	2749.43 ± 324.41	456.49 ± 100.49	1673.06 ± 408.42	2370.49 ± 676.23	186.04 ± 109.80	12,360.24
	889 (PL)	68.34 ± 7.85	1541.54 ± 198.10	717.36 ± 84.59	nd	nd	571.73 ± 59.60	61.74 ± 6.27	nd	nd	39.81 ± 6.27	nd	288.67 ± 40.08	nd	183.17 ± 67.96	nd	335.74 ± 95.02	nd	3808.1
Min		59.29	101.07	41.63	49.36	0.00	63.82	0.00	0.00	0.00	0.00	0.00	262.25	0.00	108.27	0.00	259.74	0.00	
Max		304.12	1739.98	839.53	439.35	421.47	638.47	146.71	460.84	187.68	700.08	765.78	1143.39	3856.90	1986.22	2202.94	3369.82	391.47	
Mean		182.45	559.26	346.79	195.23	316.39	155.74	51.33	129.71	92.91	268.11	459.64	656.94	2461.03	966.48	446.78	2005.30	121.32	
SD		71.77	425.86	254.38	118.76	172.67	109.42	41.75	121.35	60.73	192.82	226.22	264.33	954.23	563.96	839.08	903.22	122.06	
Variability [%]		38.98	76.15	73.35	60.83	54.57	70.26	81.35	93.55	65.37	71.92	49.22	40.24	38.77	58.35	187.81	45.04	100.61	
F-value		45,535	54.18	146.09	138.91	207.55	93.29	143.68	257.60	86.73	82.76	117.80	84.66	47.10	113.15	148.11	41.62	44.09	
*p*-value		0.000	0.000	0.000	0.000	0.000	0.000	0.000	0.000	0.000	0.000	0.000	0.000	0.000	0.000	0.000	0.000	0.000	

nd—not detected. Data are presented as mean ± SD (*n* = 3).

**Table 3:** Inhibition effect of EEP (diameter in mm) in tested concentrations on bacteria isolated from human faeces.

Sample	EEP Concentration	*Bifidobacterium* spp.	*L. rhamnosus*	*L. acidophilus*	*E. coli*	*Bacteroides* spp.
891 (BG)	70 mg/mL	16.3 ± 0.5 ^c^	18.3 ± 05 ^d^	28.3 ± 0.5 ^e^	12.3 ± 0.5 ^b^	8.3 ± 0.5 ^a^
	40 mg/mL	13.7 ± 0.5 ^c^	16.3 ± 0.5 ^d^	23.7 ± 0.5 ^e^	10.3 ± 0.5 ^b^	8.3 ± 0.5 ^a^
	10 mg/mL	11.7 ± 0.5 ^b^	15.7 ± 0.5 ^c^	20.3 ± 0.5 ^d^	7.7 ± 0.5 ^a^	7.3 ± 0.5 ^a^
	1 mg/mL	8.7 ± 0.5 ^bc^	7.7 ± 0.5 ^b^	9.3 ± 0.5 ^c^	nd	nd
	100 μg/mL	nd	nd	nd	nd	nd
	10 μg/mL	nd	nd	nd	nd	nd
885 (TR)	70 mg/mL	14.3 ± 0.5 ^d^	18.3 ± 0.5 ^a^	18.3 ± 0.5 ^a^	12.3 ± 0.5 ^c^	10.7 ± 0.5 ^b^
	40 mg/mL	11.7 ± 0.5 ^b^	15.7 ± 0.5 ^c^	14.3 ± 0.5 ^c^	10.3 ± 0.5 ^ab^	9.7 ± 0.5 ^a^
	10 mg/mL	9.7 ± 0.5 ^b^	13.7 ± 0.5 ^d^	11.7 ± 0.5 ^c^	7.7 ± 0.5 ^a^	7.7 ± 0.5 ^a^
	1 mg/mL	8.3 ± 0.5 ^a^	7.7 ± 0.5 ^a^	7.7 ± 0.5 ^a^	nd	7.3 ± 0.5 ^a^
	100 μg/mL	nd	nd	nd	nd	nd
	10 μg/mL	nd	nd	nd	nd	nd
886 (PL)	70 mg/mL	14.3 ± 0.5 ^c^	16.7 ± 0.5 ^a^	12.3 ± 0.5 ^b^	24.3 ± 0.5 ^d^	16.3 ± 0.5 ^a^
	40 mg/mL	13.7 ± 0.5 ^a^	15.7 ± 0.5 ^b^	12.3 ± 0.5 ^a^	20.3 ± 0.5 ^c^	12.3 ± 0.5 ^a^
	10 mg/mL	11.7 ± 0.5 ^a^	13.7 ± 0.5 ^b^	10.3 ± 0.5 ^a^	14.3 ± 0.5 ^b^	10.3 ± 0.5 ^a^
	1 mg/mL	8.3 ± 0.5 ^a^	8.7 ± 0.5 ^a^	nd	7.7 ± 0.5 ^a^	7.7 ± 0.5 ^a^
	100 μg/mL	nd	7.3 ± 0.5 ^a^	nd	nd	7.3 ± 0.5 ^a^
	10 μg/mL	nd	nd	nd	nd	nd
889 (PL)	70 mg/mL	18.3 ± 0.5 ^a^	9.3 ± 0.5 ^b^	18.3 ± 0.5 ^a^	12.7 ± 0.5 ^c^	16.3 ± 0.5 ^d^
	40 mg/mL	14.7 ± 0.5 ^a^	7.7 ± 0.5 ^b^	14.3 ± 0.5 ^a^	12.3 ± 0.5 ^d^	10.3 ± 0.5 ^c^
	10 mg/mL	13.7 ± 0.5 ^c^	7.3 ± 0.5 ^a^	11.7 ± 0.5 ^b^	11.3 ± 0.5 ^b^	8.3 ± 0.5 ^a^
	1 mg/mL	7.3 ± 0.5 ^b^	nd	7.3 ± 0.5 ^b^	nd	nd
	100 μg/mL	nd	nd	nd	nd	nd
	10 μg/mL	nd	nd	nd	nd	nd
893 (RO)	70 mg/mL	10.7 ± 0.5 ^a^	12.3 ± 0.5 ^b^	16.3 ± 0.5 ^c^	10.3 ± 0.5 ^a^	12.7 ± 0.5 ^b^
	40 mg/mL	9.7 ± 0.5 ^b^	11.7 ± 0.5 ^a^	12.3 ± 0.5 ^a^	8.3 ± 0.5 ^b^	11.7 ± 0.5 ^a^
	10 mg/mL	9.7 ± 0.5 ^a^	10.3 ± 0.5 ^a^	9.7 ± 0.5 ^a^	6.7 ± 0.5 ^b^	9.7 ± 0.5 ^a^
	1 mg/mL	7.7 ± 0.5 ^b^	nd	nd	nd	nd
	100 μg/mL	nd	nd	nd	nd	nd
	10 μg/mL	nd	nd	nd	nd	nd
895 (UA)	70 mg/mL	14.3 ± 0.5 ^c^	16.3 ± 0.5 ^d^	10.3 ± 0.5 ^a^	12.3 ± 0.5 ^b^	10.3 ± 0.5 ^a^
	40 mg/mL	13.7 ± 0.5 ^d^	13.7 ± 0.5 ^d^	8.3 ± 0.5 ^a^	10.3 ± 0.5 ^c^	9.7 ± 0.5 ^ab^
	10 mg/mL	11.7 ± 0.5 ^b^	11.7 ± 0.5 ^b^	7.3 ± 0.5 ^a^	8.3 ± 0.5 ^a^	7.7 ± 0.5 ^a^
	1 mg/mL	7.7 ± 0.5 ^b^	8.3 ± 0.5 ^b^	nd	nd	nd
	100 μg/mL	nd	nd	nd	nd	nd
	10 μg/mL	nd	nd	nd	nd	nd

^a,b,c,d,e^—significant differences (*p* < 0.05) between strains with the same concentration of EEP. nd—not determined. Data are presented as mean ± SD (*n* = 3).

## Data Availability

The data presented in this study are available in the article.
